# Varying Architecture of Heat Shock Elements Contributes to Distinct Magnitudes of Target Gene Expression and Diverged Biological Pathways in Heat Stress Response of Bread Wheat

**DOI:** 10.3389/fgene.2020.00030

**Published:** 2020-02-04

**Authors:** Peng Zhao, Sidra Javed, Xue Shi, Bingjin Wu, Dongzhi Zhang, Shengbao Xu, Xiaoming Wang

**Affiliations:** State Key Laboratory of Crop Stress Biology for Arid Areas, College of Agronomy, Northwest A&F University, Yangling, China

**Keywords:** bread wheat, heat stress response, heat shock elements, heat shock transcription factor, heat-responsive transcriptomes

## Abstract

The heat shock transcription factor (HSF) binds to cis-regulatory motifs known as heat shock elements (HSEs) to mediate the transcriptional response of HSF target genes. However, the HSF–HSEs interaction is not clearly understood. Using the newly released genome reference sequence of bread wheat, we identified 39,478 HSEs (95.6% of which were non-canonical HSEs) and collapsed them into 30,604 wheat genes, accounting for 27.6% wheat genes. Using the intensively heat-responsive transcriptomes of wheat, we demonstrated that canonical HSEs have a higher propensity to induce a response in the closest downstream genes than non-canonical HSEs. However, the response magnitude induced by non-canonical HSEs was comparable to that induced by canonical HSEs. Significantly, some non-canonical HSEs that contain mismatched nucleotides at specific positions within HSEs had a larger response magnitude than that of canonical HSEs. Consistently, most of the HSEs identified in the promoter regions of heat shock proteins were non-canonical HSEs, suggesting an important role for these non-canonical HSEs. Lastly, distinct diverged biological processes were observed between genes containing different HSE types, suggesting that sequence variation in HSEs plays a key role in the evolution of heat responses and adaptation. Our results provide a new perspective to understand the regulatory network underlying heat responses.

## Introduction

Wheat (*Triticum aestivum* L.), a globally important crop, contributes about a fifth of the total calories consumed by humans (IWGSC, 2018). Increasing temperatures [heat stress (HS)], especially during the grain-filling stage, adversely affect the growth and development of wheat and causes a severe reduction in its yield and quality (Tack et al., 2015; Tao et al., 2015; Lesk et al., 2016; Zhao et al., 2017; Wang et al., 2018a). Thus, the identification of thermotolerant genes and the characterization of molecular mechanisms underlying HS responses and adaptations became urgent to improve wheat thermotolerance.

Heat shock proteins (HSPs), which were first identified based on their up-regulation during heat shock, play vital roles in HS responses by assisting in protein folding and preventing irreversible protein aggregation as chaperones (Waters, 2013). Under HS, heat shock factors (HSF), which converge the heat signaling transduced from several pathways and are regarded as the terminal link in heat signaling, bind to each other to form polymers and activate the expression of HSPs by recognizing and binding to conserved DNA sequences, known as heat shock elements (HSEs), in the promoter region of HSPs (von Koskull-Doring et al., 2007; Saidi et al., 2011; Bokszczanin et al., 2013; Vu et al., 2019). In many higher eukaryotes, HSFs are a diverged gene family, with family members varying in the stimuli needed for their activation, their affinity for HSEs, and the downstream targets (Takemori et al., 2009; Tian et al., 2010; Wang et al., 2018b). However, the factors that affect this stimulus and the corresponding activation efficiency are largely unknown.

The DNA binding domain of HSFs is highly conserved, implying that sequence variations in HSEs may be primarily responsible for the varying affinity in the HSF–HSEs interaction (Tian et al., 2010; Wang et al., 2018b). Canonical HSEs comprise at least three continuous inverted repeats of the pentanucleotide sequence, 5′-NGAAN-3′, alternating between 5′-NGAAN-3′ and 5′-NTTCN-3′, or vice versa, where N is any nucleotide. The “G” at the 2nd position of the 5′-NGAAN-3′ sequence and the “C” at the 4th position of the 5′-NTTCN-3′ sequence are key nucleotides and are the most critical for the binding of HSF. Each pentanucleotide sequence was defined as a subunit that was capable of binding one monomer of the HSF trimer. In *Drosophila*, the alternating subunits, the subunit number, and the position of HSEs within the promoter correlated with the HSF affinity and the magnitude of the HS response (Xiao et al., 1991; Fernandes et al., 1995; Tian et al., 2010).

Significantly, the 3rd and 4th positions of the 5′-NGAAN-3′ sequence and the 2nd and 3rd positions of the 5′-NTTCN-3′ sequence had lower effects on the HSF–HSEs interaction, allowing a mismatched nucleotide at these positions in the consensus sequence (Fernandes et al., 1994; Tian et al., 2010). The HSF affinity for HSEs also varies with HSE sequence variations (Santoro et al., 1998; Kremer and Gross, 2009; Jaeger et al., 2014). Besides, gapped HSEs, which contain an internal 5 bp block with little or no homology to the canonical motif flanked by canonical sequences in the proper orientation, also had a comparable binding affinity to the minimal functional HSE in *Drosophila*, as long as they exceed four subunits (Amin et al., 1988; Uffenbeck and Krebs, 2006; Tian et al., 2010). Generally, the architecture of HSEs is highly diverse in the genome of yeast, *Drosophila*, and humans, with deviations from the consensus sequence, orientation, and number of subunits that could influence the DNA binding affinity of HSF and the magnitude of target gene expression. In plants, a far larger and diverged HSF gene family was observed in our previous study (Wang et al., 2018b), implying a more complex HSF–HSEs interaction system. However, the distribution and architecture features of HSEs in the plant genome and their corresponding influence on the DNA binding affinity of HSF were not thoroughly investigated, hindering our understanding on the evolution of HSF–HSEs interactions that play key roles in the HS response.

Based on the newly released wheat genome reference sequence (IWGSC, 2018), we performed a genome-wide identification of HSEs that exhibited unexpectedly higher number of genes containing HSEs in promoter regions. Using our previous intensive time-course HS response transcriptomes of wheat flag leaves and filling grain (Wang et al., 2019), we elucidated the effects of position within the promoter and diverse architectures of HSEs on the magnitude of target gene expression under HS and showed that varying HSEs mediated different biological processes in the HS response. Our results provide a new perspective to understand the mechanisms and evolution of HS response and adaptation in plants.

## Materials and Methods

### Identification and Extraction of Promoter Sequences of Wheat Genes

In order to obtain the promoter sequence of wheat high-confidence (HC) genes, we downloaded the reference genome sequence (IWGSC RefSeq v1.0, https://urgi.versailles.inra.fr/download/iwgsc/IWGSC_RefSeq_Assemblies/v1.0/iwgsc_refseqv1.0_all_chromosomes.zip), coding sequence of HC genes, IWGSC RefSeq v1.0 annotation, (https://urgi.versailles.inra.fr/download/iwgsc/IWGSC_RefSeq_Annotations/v1.0/iwgsc_refseqv1.0_HighConf_CDS_2017Mar13.fa.zip) and genome annotation (IWGSC RefSeq v1.0 annotation, https://urgi.versailles.inra.fr/download/iwgsc/IWGSC_RefSeq_Annotations/v1.0/iwgsc_refseqv1.0_HighConf_2017Mar13.gff3.zip) of the bread wheat cultivar Chinese Spring from IWGSC (IWGSC, 2018). Next, we screened the coding sequences (CDS) that started with “ATG” in genome annotation file, recorded their position on the genome, and extracted the upstream 2,000 bp sequence of CDS start sites as the promoter sequence (if the upstream sequences were less than 2,000 bp due to incomplete assembly, the longest upstream sequence was extracted) using bedtools (v2.27.1) (Quinlan and Hall, 2010) with the “getfasta” parameter. More details of each step and the relative scripts could been download on GitHub (https://github.com/biozhp/hse).

### Genome-Wide Identification of Heat Shock Elements

At present, the definition of HSE structure in plants (Busch et al,., 2005; Guo et al,., 2008) and in *Drosophila* (Tian et al., 2010) are same. Furthermore, to investigate whether the HSE motif was conserved between *drosophila* and wheat, we downloaded the HSEs of *Drosophila* and Hymenoptera insects (ant, bee, aphid, etc.) from the published article (Tian et al., 2010; Nguyen et al., 2016) and retrieved the HSEs of *Arabidopsis thaliana* from JASPAR database (http://jaspar.genereg.net/). Then we performed the multiple motif alignment among HSE derived from above species and wheat with R package “MotifStack” (Ou et al., 2018) (**Supplemental Figures 1** and **2**). The results showed that the HSEs sequences of all species actually exhibited continuous inverted repeats of nGAAn as the HSE definition. More importantly, the HSEs in wheat and *A. thaliana* were located on different clades on the phylogenetic tree, and the HSEs of non-plant species did not clustered into single clade. These results demonstrated that the HSEs between plant and no-plant species were actually conserved, without obvious divergence. Therefore, we referred to the HSEs search procedure in *Drosophila* to identify HSE in wheat.

We developed a new search procedure referred to Tian et al. (2010), as following: (1) First, we identified typical HSEs comprising at least three continuous inverted repeats of the pentanucleotide sequence 5′-NGAAN-3′, alternating between 5′-NGAAN-3′ and 5′-NTTCN-3′ or vice versa, where N is any nucleotide; each pentanucleotide sequence was defined as a subunit. (2) For the sequence-varied HSEs, a total of one nucleotide was allowed to incur in a mismatch. (3) The nucleotides “G” and “C” in the subunit of 5′-NGAAN-3′ and 5′-NTTCN-3′, respectively, were key nucleotides. (4) The key nucleotides of the first and third subunits were not allowed to incur in a mismatch when the number of subunits was three, whereas the key bases were allowed to incur in a mismatch in every subunit when the number of subunits was more than three. Furthermore, we divided sequence-varied HSEs into gapped HSEs (contain a mismatched nucleotide at the “G” or “C” position of the middle subunit) and varied HSEs (contain a mismatched nucleotide that was not at the “G” or “C” position in the middle subunit). According to the criterion of HSEs, we designed a python program to identify HSEs in the promoters of wheat HC genes. The source scripts are available at GitHub (https://github.com/biozhp/hse).

### Identification of Heat-Responsive Genes and Response Magnitude

In our previous study, wheat plants (*T. aestivum* cv. Chinese Spring) were first grown in a greenhouse under normal conditions and the plants at 15 days after anthesis were treated by heat stress (37°C) in growth chambers. The filling grain and flag leaves at 0, 5, 10, 30 min, 1, and 4 h under heat stress were harvested and subjected to 150 bp paired-end sequencing using the Illumina HiSeq X Ten platform. With the sample at 0 min time point which were not treated by HS as control, the differentially expressed genes (fold change ≥ 2.0 and false discovery rate-adjusted *p* < 0.05) at each heat stress treatment time point were identified (Wang et al., 2019). Genes that were differentially expressed at any HS treatment time point were defined as heat-responsive genes in each organism in the present analysis. Then, we investigated the maximal fold changes of heat-responsive genes among five time points (5, 10, 30 min, 1, and 4 h) under HS in each organism and normalized the maximal fold changes using the function of “scale (center = T, scale = T)” in R program. Finally, the normalized maximal fold change was used as an indicator to reflect the responsive magnitude of closest downstream genes (CDGs).

### Gene Ontology Enrichment Analysis

The gene ontology (GO) annotation was obtained from our previous study (**Supplemental Table 1**) (https://zenodo.org/record/2541477/files/Genes_transcripts_FPKM.zip) (Wang et al., 2019). We used the R package “clusterProfiler” with the “enricher” function for enrichment analysis (Yu et al., 2012). The statistical significance of the GO enrichment was examined using the hypergeometric distribution test, followed by multiple-test correction using the Benjamini–Hochberg method (Benjamini and Hochberg, 1995). GO terms with *q* < 0.01 were retained for further analysis. The source codes and input files are available at GitHub (https://github.com/biozhp/hse/tree/master/example/enrichment).

### Statistical Analysis

All statistical analyses were performed using the R-3.6.1. The function “chisq.test” with argument “correct = FALSE” was used for Pearson’s Chi-squared test. One-way analysis of variance (one-way ANOVA) was performed with the function “aov” Multivariate analysis of variance (MANOVA) was performed with the function “manova” Multiple comparison analysis was performed with the R package “multcomp”. The used source codes and input files are available at GitHub (https://github.com/biozhp/hse/blob/master/example/statistics.R).

### Motif Enrichment Analysis

We performed motif enrichment analysis in the promoter regions of heat stress response genes and no heat stress response genes with the AME program (http://meme-suite.org/tools/ame) from MEME package (McLeay and Bailey, 2010), using the JASPAR CORE 2018 database as background. Then select motif with p-value < 0.01, q-value < 0.05 and e-value < 1e-5 for further analysis.

## Results

### Identification of Heat Shock Elements in the Wheat Genome

**Figure 1:**
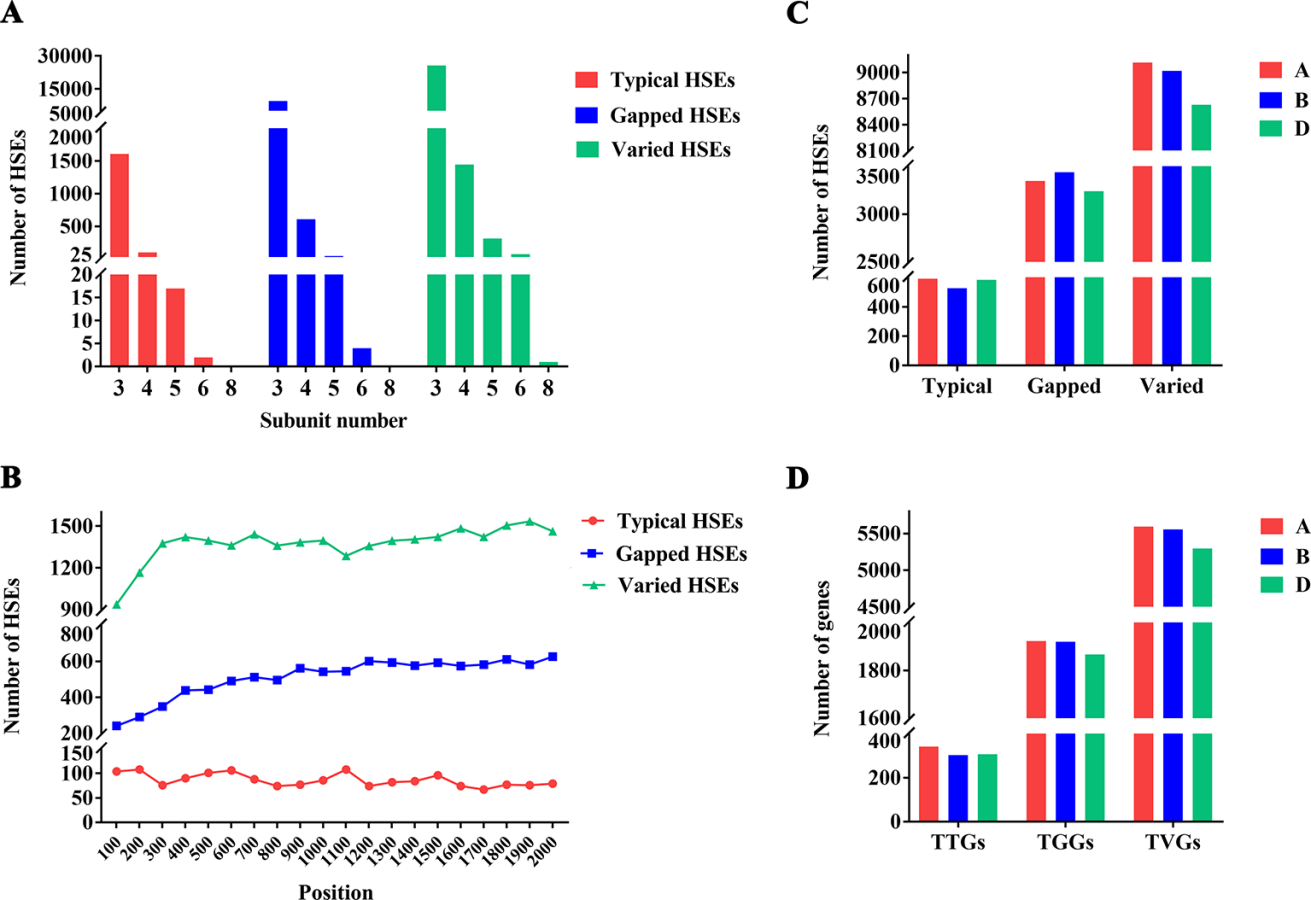
Distribution of HSEs and its CDGs in wheat genome **(A)** Number of different HSE types. The x-axis represents the subunits and the y-axis represents the number of HSEs. Red, blue, and green represent the number of typical HSEs, gapped HSEs, and varied HSEs, respectively. **(B)** Positions of different types of HSEs within promoters. The x-axis represents the position. The “0” indicates the “TSS” and the “2000” indicates the upstream 2000th bp of TSS. The y-axis represents the number of HSEs. Red, blue, and green represent the number of typical HSEs, gapped HSEs, and varied HSEs, respectively. **(C)** Distribution of HSEs among three wheat subgenomes. The x-axis represents the different types of HSEs and the y-axis represents the number of HSEs. Red, blue, and green represent the **(A, B, D)** subgenome, respectively. **(D)** Distribution of genes containing different types of HSEs among three wheat subgenomes. The x-axis represents the genes containing different types of HSEs and the y-axis represents the number of genes. Red, blue, and green represent the A, B, and D subgenome, respectively.

Based on the fact that the HSE motif was conserved between *drosophila* and bread wheat (see “Methods”), we defined the wheat HSE identification criterion referring to the definition for HSEs in *Drosophila* (Tian et al., 2010). The genome of the bread wheat cultivar Chinese Spring exhibited 39,478 computationally identifiable HSEs in the promoter regions of all HC genes (**Supplemental Tables 2** and **5**), including 1,727 typical/canonical HSEs (three or more canonical 5 bp subunit sequences of 5′-NGAAN-3′ and 5′-NTTCN-3′ in alternation), 10,234 gapped HSEs (contain a mismatched nucleotide at the “G” or “C” position of the middle subunit), and 27,517 varied HSEs (contain a mismatched nucleotide that was not at the “G” or “C” position in the middle subunit) (**Supplemental Figure 3**). Unexpectedly, the number of varied and gapped HSEs was much larger than that of typical HSEs (**Figure 1A**), making it intriguing whether varied and gapped HSEs have the ability to interact with HSF to induce the expression of CDGs. Moreover, the subunit number of identified HSEs ranged from three to eight, and the number of all types of HSEs sharply decreased while the subunit number increased (**Figure 1A**). For the positions of HSEs in promoters, all HSE types were evenly distributed within the promoters (**Figure 1B**). Afterward, the distribution of HSEs among three wheat subgenomes was uneven, with the largest and smallest numbers of typical and varied HSEs on the A subgenome and the gapped HSEs on the D subgenome, respectively (**Figure 1C**). These results demonstrated that the wheat genome contains massive HSEs and exhibits evolutionary divergence for the subunit number, position, and sequence conformity.

The identified HSEs were collapsed into the promoter regions of 30,604 genes (27.62% of all HC genes annotated in IWGSC RefSeq 1.0). For these genes, 968, 5,816, and 16,906 genes contained only one typical HSE, gapped HSEs, or varied HSEs in their promoter regions and were designed as TTGs (Genes contained only one typical HSEs), TGGs (Genes contained only one gapped HSEs), and TVGs (Genes contained only one varied HSEs), respectively. The remaining 6,914 genes contained more than one HSEs in their promoter regions. The TTGs, TGGs, and TVGs were also unevenly distributed among three wheat subgenomes, with the largest number of TTGs, TGGs, and TVGs in the A subgenomes (**Figure 1D**). For clarity and accuracy, only TTGs, TGGs, and TVGs were used in the following analysis.

### Varying Architecture of Heat Shock Elements Affect Heat Response of Closest Downstream Genes

To understand how the different types of HSEs affect the expression of CDGs, we comprehensively investigated the expression fold change of TTGs, TGGs, and TVGs under HS, which is a well-known inducing factor of HSF binding to HSEs; these, in turn, activate the expression of target genes, using the heat-responsive transcriptomes of wheat flag leaves and filling grain under HS at 0 min, 5 min, 10 min, 30 min, 1 h, and 4 h, as reported in our previous study (Wang et al., 2019). In filling grain, a total of 33 TTGs (3.4%), 75 TGGs (1.3%), and 339 TVGs (2.0%) responded to HS, compared to the untreated samples (fold change ≥ 2.0 and false discovery rate-adjusted *p* < 0.05), accounting for 1.6%, 3.5% and 15.9%, respectively, of the grain heat-responsive genes identified in our previously study. Similarly, in flag leaves, the relative numbers were 108 TTGs (11.2%), 301 TGGs (5.2%), and 1,212 TVGs (7.2%), accounting for 1.6%, 4.4% and 17.5%, respectively, of the heat-responsive genes in flag leaves. As expected, TTGs had a significantly higher proportion to response to HS than TGGs and TVGs in grain (Pearson’s Chi-squared test, *X^2^* = 24.6, *p* = 4.5E-06) and in leaves (*X^2^* = 56.5, *p* = 5.5E-13), implying that the typical HSEs have higher abilities or affinities for binding to HSFs. These results demonstrated that the presence of HSEs was not equal to the HS response, and that HSEs sequence variations also affected this response, suggesting a more complex network and mechanism of HSF in mediating a HS response.

**Figure 2:**
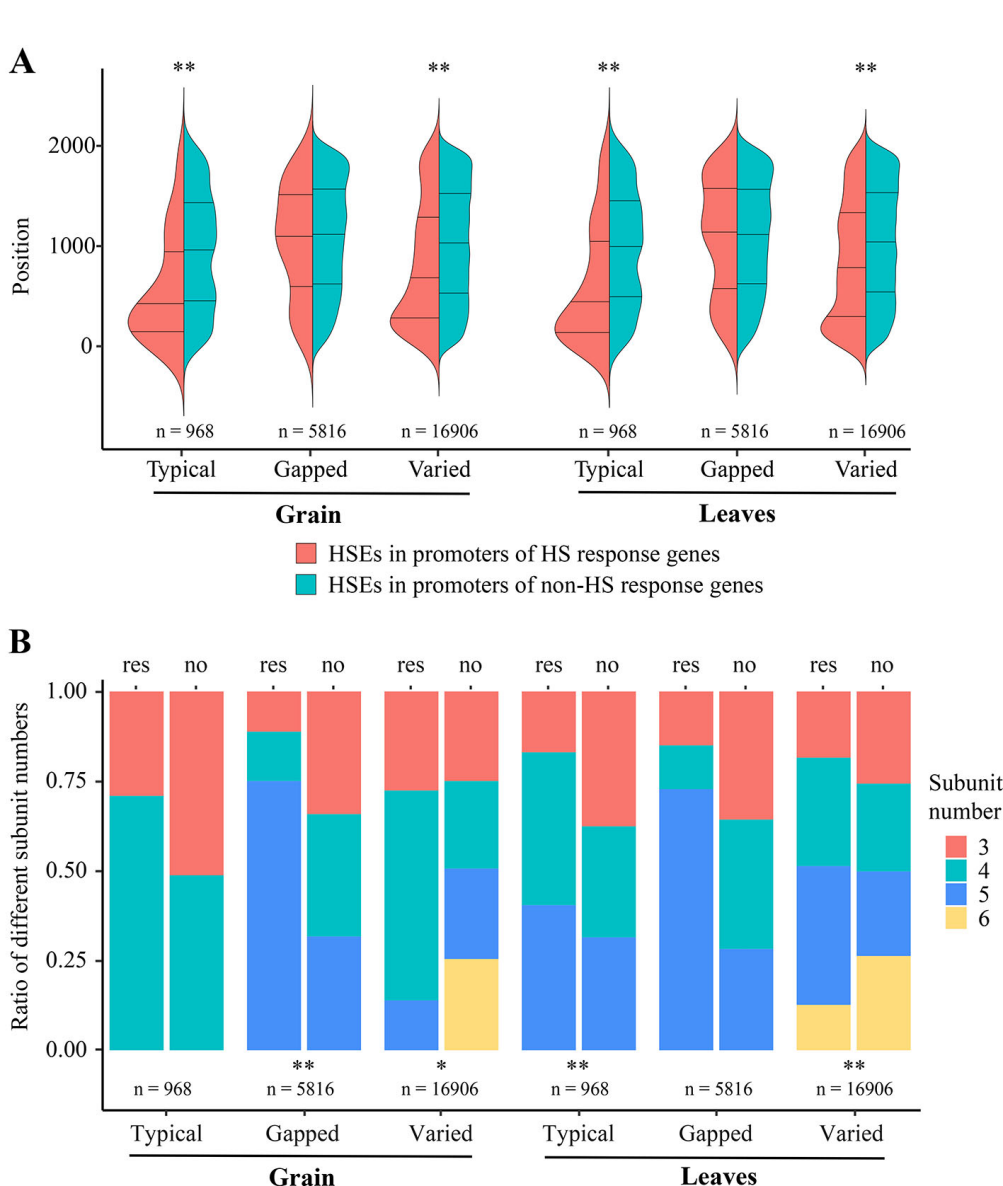
Effects of varying architecture in different HSE types on the HS response of CDGs **(A)** HSE positions within promoters. The x-axis represents the different types of HSEs. The y-axis represents the positions of HSEs within promoters and the “0” indicates the “ATG”. Red represents the HSEs in promoters of HS response genes, green represent the HSEs in promoters of non-HS response genes. **(B)** Ratio of different subunit numbers of HSEs. The x-axis represents the different types of HSEs and the y-axis represents the ratio of genes containing different subunit number of HSEs to all genes containing each type of HSEs. The “res” and “no” indicate the HS response gene set and the non-HS response gene set, respectively. Red, green, blue, and yellow represent the HSEs with 3, 4, 5, 6 subunits, respectively. * and ** represent the significant difference of p-value < 0.05 and p-value < 0.01, respectively.

In detail, to further understand why some of the TTGs, TGGs, and TVGs do not respond to HS, we analyzed the effects of position within promoters and subunit number of HSEs on the response of CDGs to HS, using a MANOVA. The results showed that these two factors significantly affect the responses of TTGs in leaves and TVGs in grain and leaves (**Table 1**). Interestingly, the largest effects were observed on the response of TVGs in both grain and leaves, implying that position and subunit number of HSEs could compensate for the adverse effects of mismatched nucleotides in HSEs to some extent. Generally, a higher proximity of HSEs and TSS resulted in a higher ratio of CDGs that responded to HS, with the exception of TGGs (**Figure 2A**). For the subunit number, the HSE subunits four and five accounted for most of the HS responsiveness in HSEs (**Figure 2B**). The above results indicated that the architecture of HSEs affected whether CDGs respond to HS, suggesting that the HS response of a certain gene can be modulated by modifying the structure of HSEs in the promoter sequence.

**Table 1:** The effects of position within promoters and subunit number of HSEs in genes response to HS using multivariate analysis of variance.

	Grain			Leaves		
	TTGs	TGGs	TVGs	TTGs	TGGs	TVGs
*F-value*	7.697	3.705	25.589	25.315	0.857	72.407
*P-value*	4.83E-04**	2.47E-02**	8.01E-12**	1.93E-11**	4.25E-01	<2.2e-16**
Positions *P-value*	1.81E-04**	6.02E-01	7.07E-12**	3.19E-10**	8.96E-01	<2.2e-16**
Subunits *P-value*	2.74E-01	7.41E-03**	3.06E-02*	2.42E-03**	8.96E-01	4.00E-06**

* and ** represent the significant difference of *p-value* < 0.05 and *p-value* < 0.01, respectively.

### Sequence-Varied Heat Shock Elements Had a Comparable Heat Stress Response Magnitude With Typical Heat Shock Elements

Given that the HSE architecture affects whether CDGs respond to HS, it is intriguing whether this architecture affects the HS responsive magnitude. To understand this relationship, we used the normalized maximal fold change (MFH) among five time points (5 min, 10 min, 30 min, 1 h, and 4 h) under HS in each organism as an indicator of the responsive magnitude of CDGs. The MFH of all HS-responsive TTGs, TGGs, and TVGs was first illustrated and no significant differences were observed among the three gene types in either grain (One-way ANOVA, *p* = 0.143) or leaves (*p* = 0.08) (**Supplemental Figure 4**), suggesting that the HS response magnitude of gapped and varied HSEs was comparable for CDGs with typical HSEs, although typical HSEs had a higher propensity to induce CDGs in response to HS. These results are consistent with the fact that almost all of the HSEs in the promoter regions of HSP genes, which are well-known target genes of HSF and marker genes in the HS response due to their sharply up-regulated expression (Waters, 2013), were varied HSEs (**Supplemental Figures 5–8**).

Furthermore, the relationship between HSE architecture (the position within promoter and the subunit number) and the responsive magnitude of each HSE type was investigated. Results showed that upon higher proximity of HSEs to TSS, a higher response magnitude of TVGs in grain, and TGGs and TVGs in leaves (**Tables 2** and **3**). The subunit number only significantly affects the responsive magnitude of TVGs in leaves, for which the four HSE subunits confer a higher response magnitude (**Supplemental Figure 9**). These results demonstrated that sequence variations in HSEs (HSEs type) did not affect the HS responsive magnitude of CDGs, whereas the HSEs architecture indeed contributes to this magnitude in each HSE type.

**Table 2:** The effects of position within promoters and subunit number of HSEs in genes response magnitude using analysis of covariance.

Tissues	Type	Variates	*F-value*	*P-value*
Grain	TTGs	Positions	0.478	4.95E-01
		Subunits	1.071	3.09E-01
	TGGs	Positions	0.155	6.95E-01
		Subunits	1.304	2.57E-01
	TVGs	Positions	5.744	1.71E-02*
		Subunits	1.910	1.68E-01
Leaves	TTGs	Positions	1.580	2.12E-01
		Subunits	0.018	8.94E-01
	TGGs	Positions	9.143	2.71E-03**
		Subunits	0.264	6.08E-01
	TVGs	Positions	26.850	2.58E-07**
		Subunits	12.040	5.38E-04**

* and ** represent the significant difference of *p-value* < 0.05 and *p-value* < 0.01, respectively.

**Table 3:** The effects of position within promoters in genes response magnitude.

Tissues	Type	*Coefficient of correlation*	*P-value*
Grain	TVGs	-0.138	1.12E-02*
Leaves	TGGs	-0.174	2.39E-03**
	TVGs	-0.158	3.07E-08**

* and ** represent the significant difference of *p-value* < 0.05 and *p-value* < 0.01, respectively.

### Mismatched Nucleotide At a Specific Position Within Heat Shock Elements Had a Larger Heat Stress Response Magnitude

**Figure 3:**
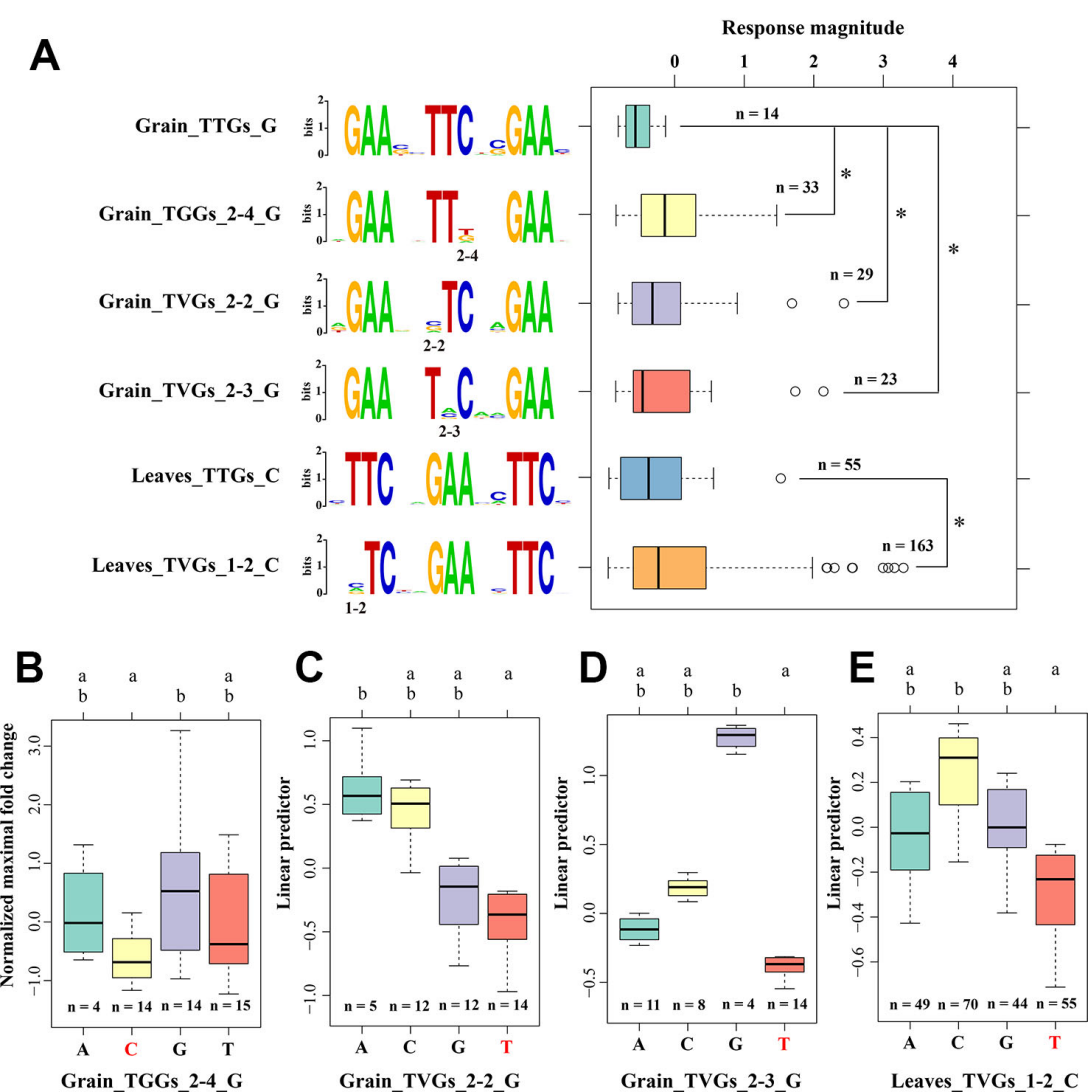
Preference for the position and nucleotide in sequence variations of HSEs **(A)** The effects of different mismatched nucleotides in HSEs on the MFH of CDGs. Compared to the response magnitude of typical HSEs using one-way analysis of covariance with the position of HSEs within the promoter as covariance. “G” and “C” represent the first subunit sequence with 5′-NGAAN-3′ and 5′-NTTCN-3′, respectively. “2-3:” represents the 3rd position in the second subunit. The sequence logo on the middle part illustrates the sequence of each HSE defined on the left. The response magnitude was represented by the normalized maximum fold change value among five heat stress treatment time point. *represent the significant difference of *p* < 0.05. **(B–E)** The effects of different mismatched nucleotides in HSEs on the MFH of CDGs using multiple comparative analysis. The letters above the boxplot represent the significance level and the data sets with different letters were significantly different (*p* < 0.05). Red bases at the x-axis represent the nucleotides of canonical HSEs at this position. Y-axis represents the linear predictor of MFH.

Although no significantly different response magnitudes were observed among the three HSE types, whether mutations on a specific position or a specific mismatched nucleotide in HSE sequences correlated with the response magnitude is an open question. First, we excluded the effects of subunit number by only focusing on the three HSE subunits that had the largest numbers in our analysis. Then, using one-way analysis of covariance with the position of HSEs within the promoter as covariance, we found four positions within HSEs at which mismatched nucleotides significantly affected the response magnitude compared with that of typical HSEs (**Figure 3A**). For instance, the mismatch on the second or third positions in the second subunit of the sequence 5′-NTTCN-3′ in grain, and the 2nd position in the first subunit of the sequence 5′-NTTCN-3′ in leaves significantly affected the response magnitude. Unexpectedly, mismatches at the four positions conferred a significantly larger response magnitude than that of canonical HSEs (**Figure 3A**), suggesting evolutionary advantages of nucleotide mutations at these positions. To further support this conclusion, massive sequence varied HSEs at these four positions were observed in the promoter regions of HSP genes, especially the HSPs that had a larger response magnitude after HS treatment (**Supplemental Figures 5–8**). It is also noteworthy that these mismatched positions were different between grain and leaves, consistent with our previous results that these two organisms exploit different molecular mechanisms and networks underlying the HS response (Wang et al., 2019).

Furthermore, we analyzed the effects of different mismatched nucleotides on these four positions using multiple comparative analyses. Different magnitudes were observed among HSEs that had different mismatched nucleotides on the same position, although some difference was not statistically significant (**Figures 3B–E**). For example, the mismatched nucleotide “A” on the 2nd position of the second subunit in the sequence 5′-NTTCN-3′ in grain had a larger response magnitude than the mismatched nucleotides “G” and “C” at the same position. These results showed that mutations in HSE sequences have preferences for position and nucleotides in the evolution of the HS response and adaptation, providing clues for the response magnitude improvement of HSEs.

### Distinctly Functional Divergence of Genes Containing Different Heat Shock Elements Types

**Figure 4:**
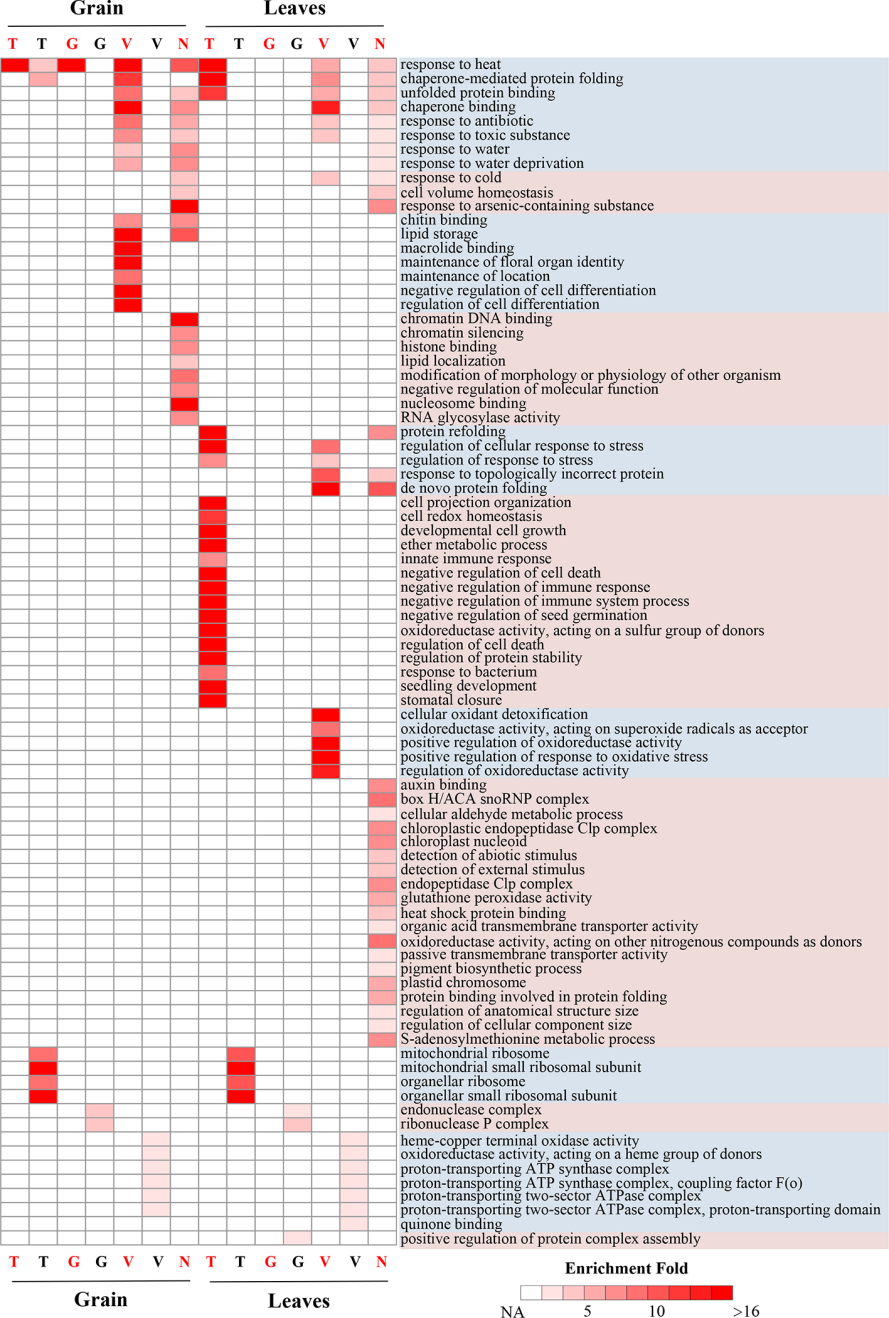
GO enrichment analysis for HS response and non-HS response of TTGs, TGGs, and TVGs GO enrichment analysis for HS response and non-HS response of TTGs, TGGs, and TVGs in grain and leaves. “T,” “G,” “V,” and “N” represent the TTGs, TGGs, TVGs, and genes that do not contain HSEs in their promoter regions, respectively. Red letters at the x-axis represent the HS-response genes. Black letters at the x-axis represent the non-HS response genes. Heat maps show the fold enrichment of enriched GO terms; only significantly enriched terms (*q* < 0.01) are indicated.

Due to the contribution of HSE architecture to the HS response of CDGs, it is intriguing whether different HSE types were involved in different biological functions and pathways in the HS response. We performed a GO enrichment analysis for HS-response and non-HS-response of TTGs, TGGs, and TVGs, and for genes that do not contain HSEs in the promoter region but respond to HS. Interestingly, genes with different HSE types showed significantly distinct GO enrichment terms (**Figure 4**), suggesting a contribution of HSE sequence variations in the evolution of HS adaptation and response. The HS-responsive TTGs was mainly over-represented in the well-known HS response terms, such as “response to heat” and “chaperonemediated protein folding” whereas the HS-responsive TVGs showed significant over-representation in terms involved in extended HS response processes, such as “chaperone binding” “regulation of cell differentiation” and “positive regulation of response to oxidative stress”. The HS-responsive TGGs was only enriched in terms of “response to heat” in grain, and had no significant enrichment terms in leaves. Combined with the previous result showing that TGGs had a lower propensity to induce an HS response of CDGs (when compared to TTGs and TVGs), we proposed that gapped HSEs tend to release from HSF regulation. It is also interesting that most of the over-represented terms of the HS-responsive genes, which contain no HSEs in their promoter regions but respond to HS, were not overlapped with terms that were enriched in genes containing HSEs; this suggests an important role for HSEs in the evolution of HS response and adaptation.

Unexpectedly, a number of GO terms were also significantly enriched in the non-HS-responsive TTGs, TGGs, and TVGs (**Figure 4**). For example, “oxidoreductase activity” and energy metabolism-related terms were enriched for non HS-responsive TVGs, and “mitochondrial ribosome,” “mitochondrial small ribosomal subunit,” and “organellar small ribosomal subunit” were enriched for non HS-responsive TTGs, posing an intriguing question as to what roles do these processes play in the evolution of HS response and adaptation. These results demonstrate that different HSE types derive distinctly diverged HS response processes and provides a new perspective for understanding the evolution of HS response and adaptation.

## Discussion

In this study, we comprehensively identified the distribution of HSEs and illustrated, for the first time, that varying HSE architecture affects the HSF DNA binding affinity and the corresponding response magnitude of CDGs in plants, thus mediating different HS response processes. Our results, including the large number of genes containing HSEs, the vast majority of varied HSEs, the comparable or higher HS response magnitude of genes containing varied HSEs, and the diverged HS response processes mediated by different HSEs types, suggest a complex interaction network in HS response and provide a new perspective to understand the HS response and adaptation.

HSEs that have mismatched nucleotides at specific positions have larger response magnitudes than that of typical HSEs. Furthermore, the diverged biological process that different HSE types are involved in, proposed an important role for HSE sequence variation in the evolution of HS response and adaptation in plants. It seems like differences in the HS response magnitude of specific genes and biological processes are the result of HSE architecture variation, instead of variations in DNA binding sequence of HSFs or the adjustment of the interacting proteins with HSFs. In our previous study, the oligomerization domain and the transcriptional activation domain of HSFs exhibited larger sequence divergences than that of DNA binding domains during plant evolution (Wang et al., 2018b), which may also contribute to the variations in HS response magnitude of target genes. Therefore, research aimed at the coevolution of HSEs and HSF will be vital for understanding the evolution of HS response and HS adaptation in plants.

It is an interesting question whether the varied HSEs were derived from mutations of typical HSE sequences or derived from evolution and natural selection of mutations from non-HSE sequences. Theoretically, the first hypothesis only needs one mutation, whereas the second hypothesis needs one or more mutations, making the first hypothesis more reasonable. However, in our results, the non-overlapped GO enrichment terms between genes containing typical HSEs and genes containing varied HSEs make the answer increasingly ambiguous. In the future, HS response analysis in more plant species, especially in ancient plants, will facilitate the answer to this question.

In our previous study, small HSPs, key genes in the HS response, maintained higher transcriptional levels in filling grains than in flag leaves (Wang et al., 2017). Furthermore, we also found that the number of HS-responsive genes, the response patterns, the involved pathways, and the responsive magnitude between filling grains and flag leaves were distinctly different (Wang et al., 2019). Here, the effects of HSE varying architecture on the response magnitude and the preference for both the position and nucleotide in HSE sequence variations were also different between these two organisms. Because the HSE DNA sequences were equal between these two organism, we assume that different factors, such as the proteins that interacted with HSFs or the transcription initiation complexes, the status of chromatin, and the energy status, resulted in the different effects observed in this study, thus highlighting the differences of gene networks exploited by grain and leaves in HS response.

It is interesting that the vast majority of TTGs, TGGs, and TVGs were not HS-responsive genes and a number of GO terms were also significantly enriched among these genes, implying that these genes and their involved processes may lose their roles in HS response owing to sequence variation of HSEs in their promoter regions or that they do not respond to HS in our investigated organism and HS treatment time points. More importantly, these results motivated us to consider the factors that interact with HSFs to discriminate between HSEs. In the data analysis, several significantly enriched motifs were observed in the promoter regions of HS-responsive genes and non HS-responsive genes, respectively (**Supplemental Tables 3** and **4**), with the AME program (http://meme-suite.org/tools/ame) from MEME package, using the JASPAR CORE 2018 database as background. Interestingly, some enriched motifs were different between the HS-responsive genes and non HS-responsive genes, providing the possibility that other transcription factors may modulate HSF binding to these promoters and affect the subsequent HSF affinity. On the other hand, in *Drosophila*, a ChIP-seq assay showed that HSFs discriminate HSEs based on local signatures of active chromatin (Guertin and Lis, 2010). In wheat, the role of chromatin status variation in HS response was also observed (Liu et al., 2015; Liu et al., 2018), implying that local chromatin status is one of the important factors affecting the interaction between HSF and HSEs. Lastly, other than HS, HSFs were also regarded as core components of signal transduction chains in various abiotic stresses and played a critical role in abiotic stresses response in plants (Guo et al., 2016; Wang et al., 2018b). Therefore, HSEs in the promoter regions of non-HS responsive genes may be discriminated and bound by HSF under other abiotic stresses.

A high efficiency promoter or DNA cis-element in HS response is important not only for the thermotolerance improvement of crops by modulation of gene expression but also for molecular biology studies, because it could be used as an inducible and sharply up-regulated promoter in gene transformation. Although we did not find a specific HSE architecture that continuously had a large response magnitude after heat shock, the finding that varying HSE architecture affects this response provides valuable clues for the directed design of promoters in the future.

## Data Availability Statement

The datasets generated for this study are available on request to the corresponding author.

## Author Contributions

PZ, XS, and BW carried out the data collection. PZ, BW, and XW performed the data analyses. SX and XW contributed to the study design. PZ and XW wrote the manuscript. All authors were involved in the revision of the manuscript and approved the final manuscript.

## Funding

This work was supported by the National Natural Science Foundation of China (31501380).

## Conflict of Interest

The authors declare that the research was conducted in the absence of any commercial or financial relationships that could be construed as a potential conflict of interest.

## Abbreviations

HSEs, Heat shock elements; CDGs, Closest downstream genes; TTGs, Genes contained only one typical HSEs; TGGs, Genes contained only one gapped HSEs; TVGs, Genes contained only one varied HSEs; TSS, Transcription start site; MFH, Normalized maximal fold change.
