# Supplementary material for: Varying Architecture of Heat Shock Elements Contributes to Distinct Magnitudes of Target Gene Expression and Diverged Biological Pathways in Heat Stress Response of Bread Wheat
Source: Front Genet. 2020 Feb 4;11:30. doi: 10.3389/fgene.2020.00030 (PMC7010933; doi:10.3389/fgene.2020.00030)
Supplement: Supplementary file 2 [file Presentation_1.pdf]

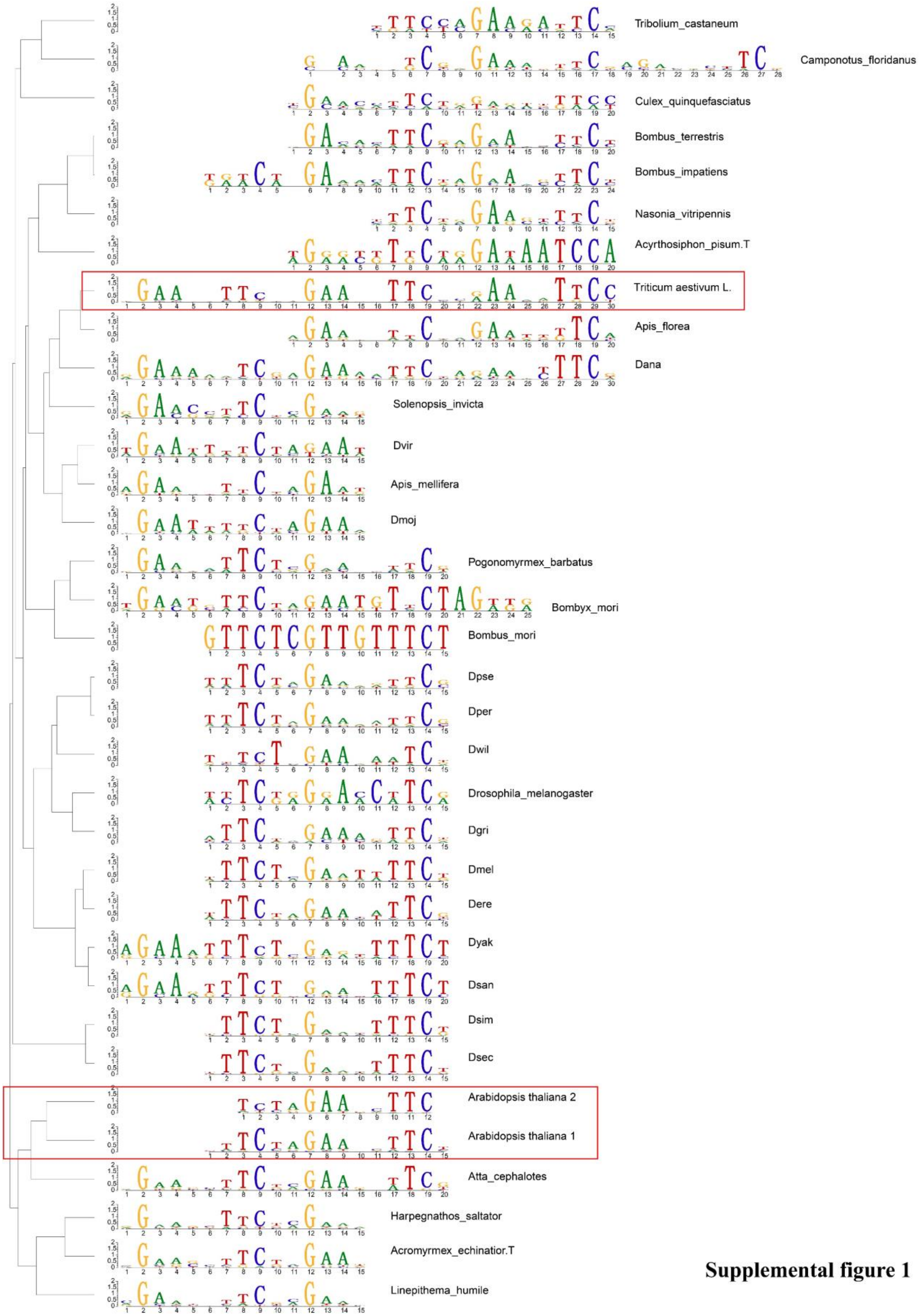

Supplementary Figure 1. The result of multiple motif alignment of HSEs that start with the subunit sequence of 5′-NGAAN-3′.

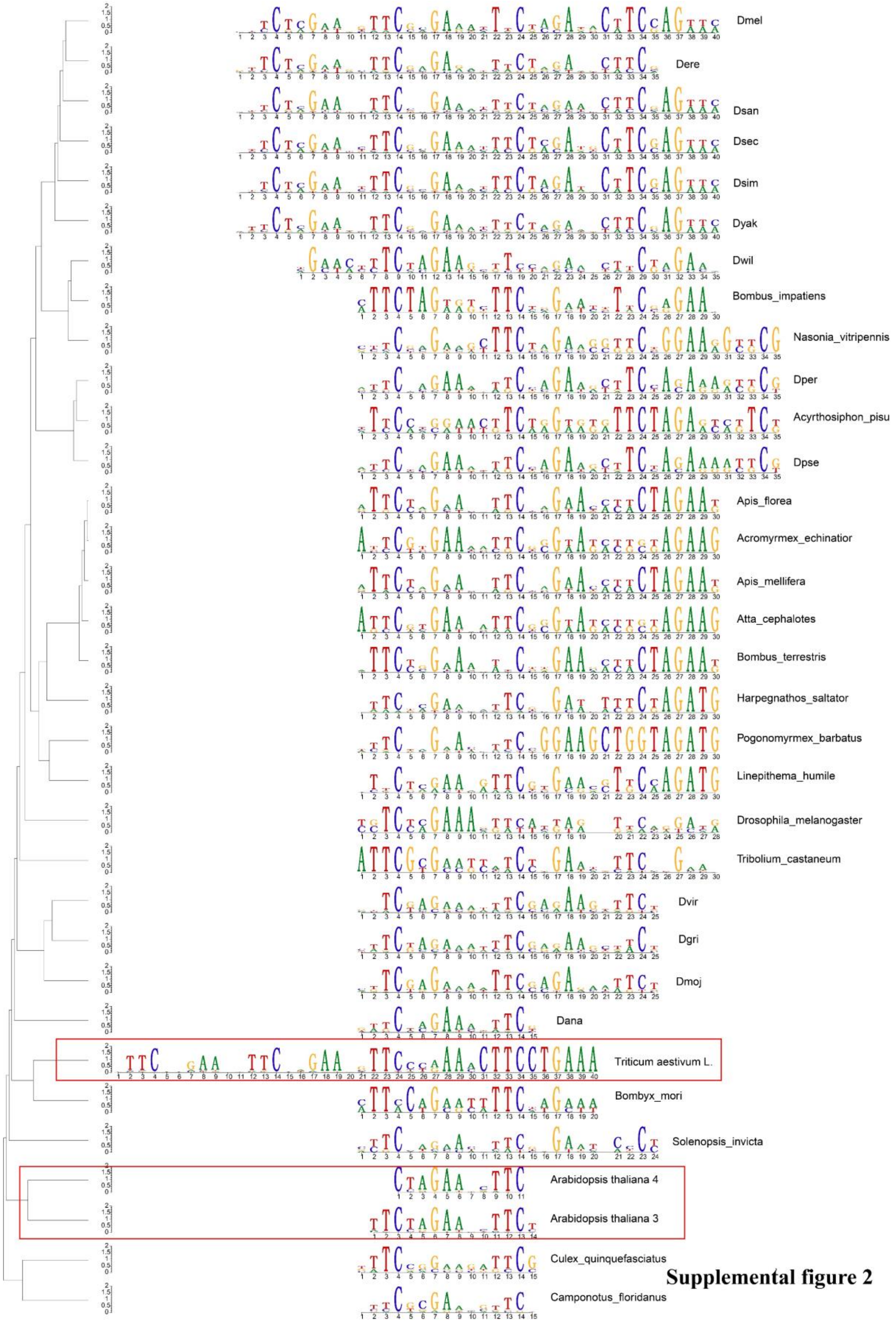

Supplementary Figure 2. The result of multiple motif alignment among HSEs that start with the subunit sequence with 5′-NTTCN-3′.

Supplementary Figure 3

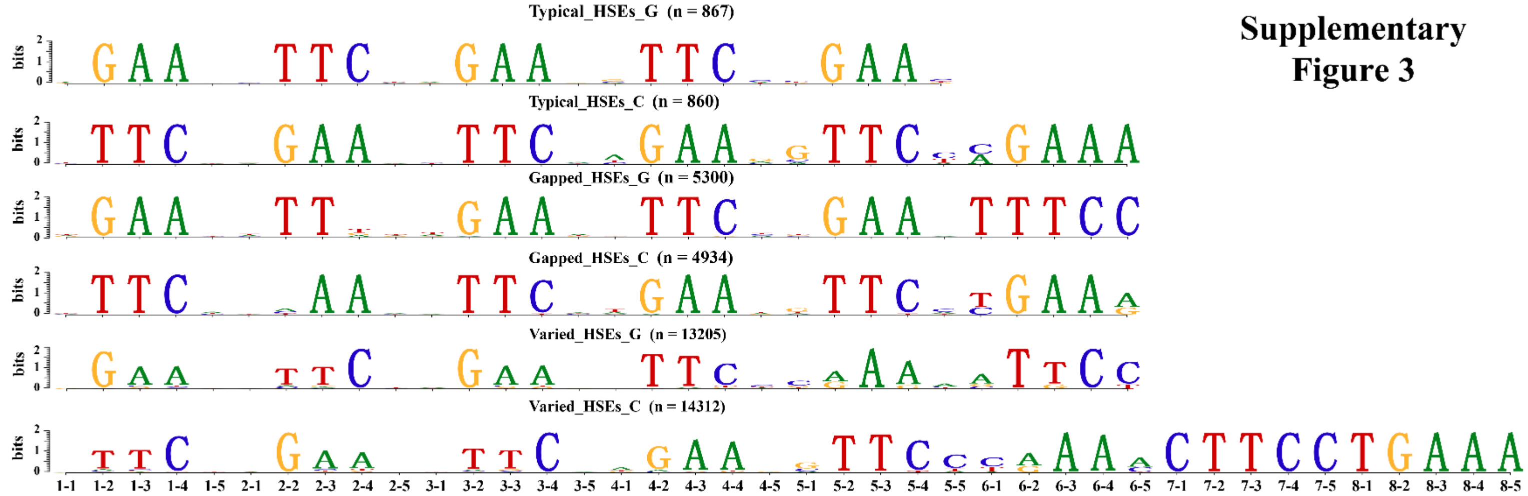

Supplementary Figure 3. The sequence logos for the typical, gapped and varied HSEs. The x-axis represents the position of nucleotides. For example, “2-3” represents the 3rd position in the second subunit. “G” and “C” in the HSE classification represent the first subunit sequence with 5′-NGAAN-3′ and 5′-NTTCN-3′, respectively.

Supplemental figure 4

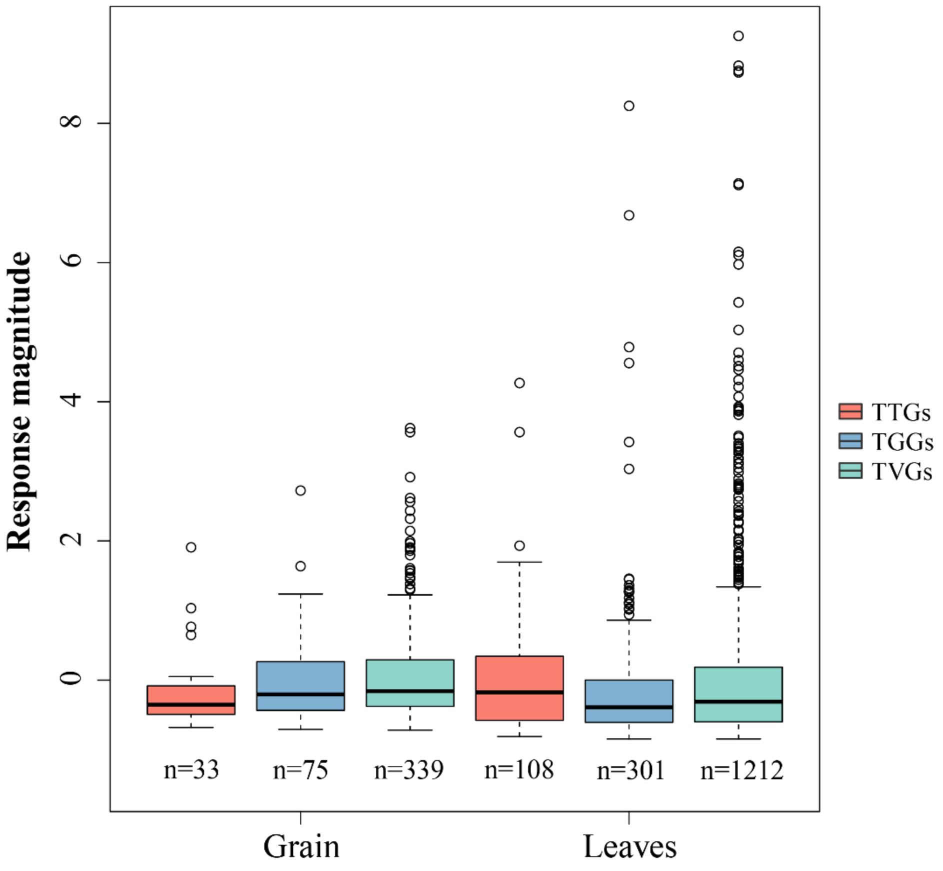

Supplementary Figure 4. The response magnitude of all TTGs, TGGs and TVGs in grains and leaves. The y-axis represents the response magnitude of genes containing different type HSEs in grains and in leaves. “n” represents the number of genes.

Supplemental figure 5

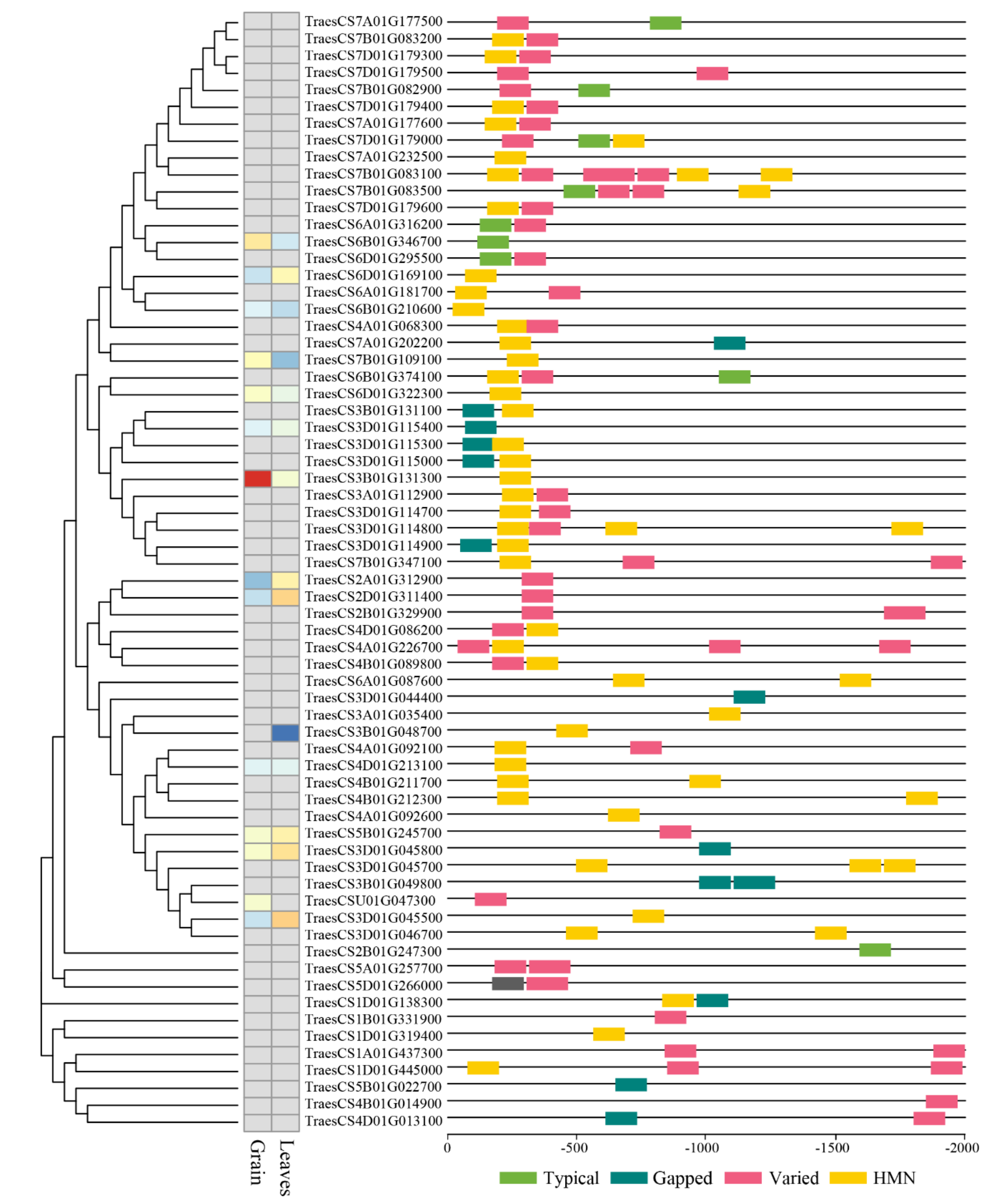

Supplementary Figure 5. The response magnitude and distribution of HSEs of HSP20 in grain and leaves.

The phylogenetic tree was generated based on the full length amino acid sequences of HSP20 with the neighbor-joining (NJ) method. The heatmaps on the left represents response magnitude of HSP20s in grain and leaves, respectively. The right part illustrate the architecture and distribution of HSEs in the promoter regions of each HSP20. One rectangle represents one HSE. For clarity, the length of the rectangle is tenfold the length of HSEs. “HMN” represents the varied HSEs that have mismatched nucleotide at specific position and have lager response magnitude than that of canonical HSEs.

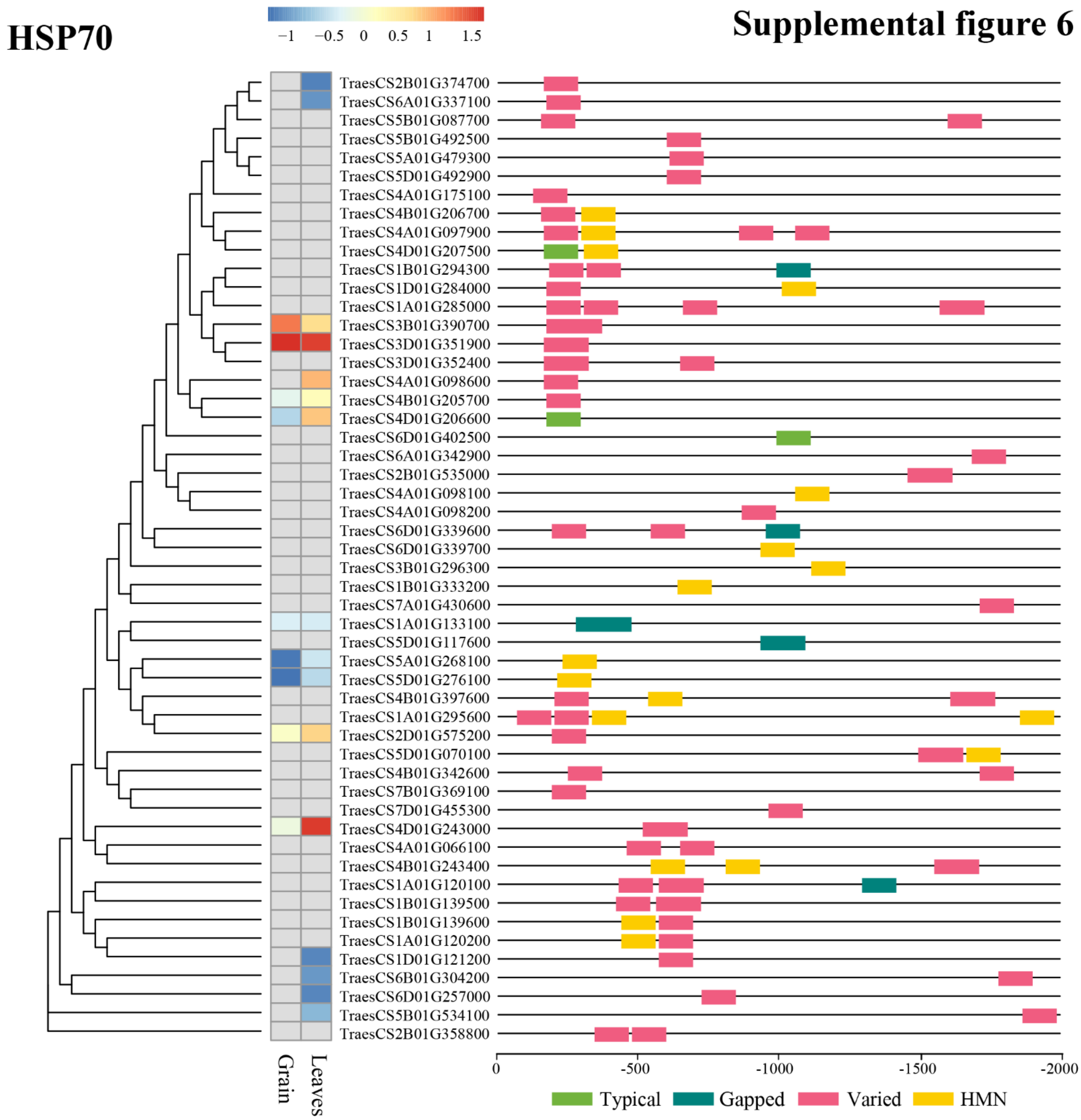

Supplementary Figure 6. The response magnitude and distribution of HSEs of HSP70 in grain and leaves.

The phylogenetic tree was generated based on the full length amino acid sequences of HSP70 with the neighbor-joining (NJ) method. The heatmaps on the left represents response magnitude of HSP70s in grain and leaves, respectively. The right part illustrate the architecture and distribution of HSEs in the promoter regions of each HSP70. One rectangle represents one HSE. For clarity, the length of the rectangle is tenfold the length of HSEs. “HMN” represents the varied HSEs that have mismatched nucleotide at specific position and have lager response magnitude than that of canonical HSEs.

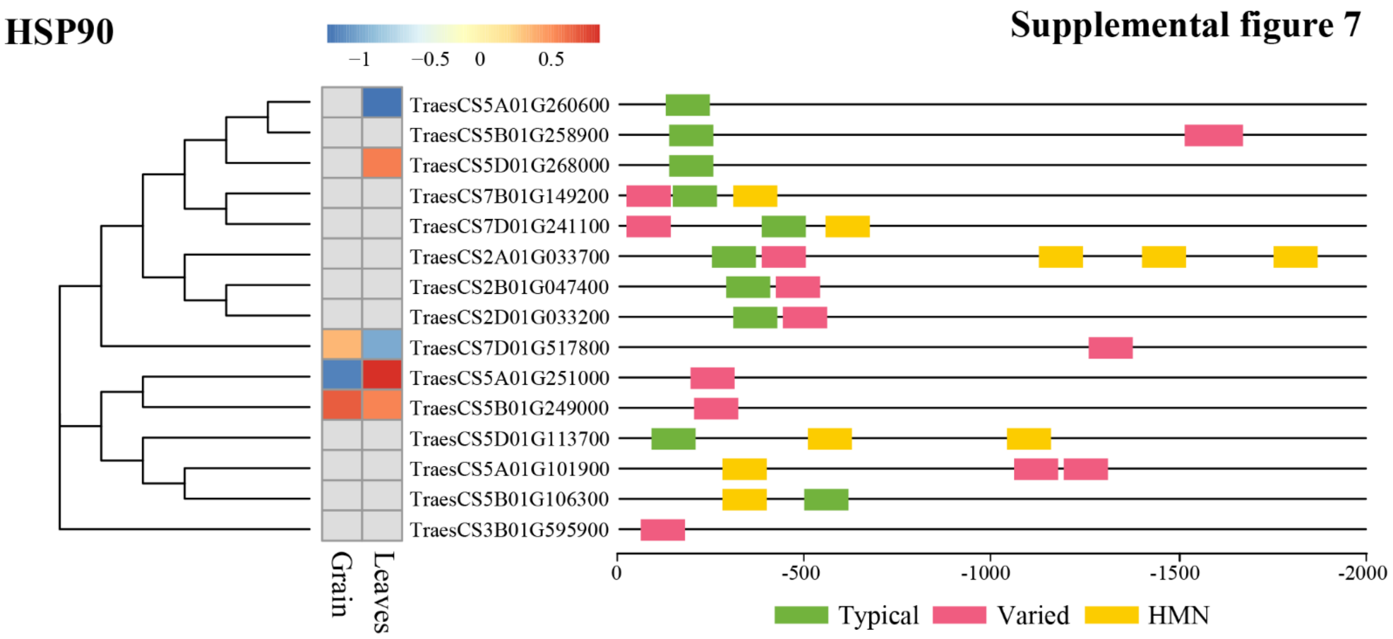

Supplementary Figure 7. The response magnitude and distribution of HSEs of HSP90 in grain and leaves.

The phylogenetic tree was generated based on the full length amino acid sequences of HSP90 with the neighbor-joining (NJ) method. The heatmaps on the left represents response magnitude of HSP90s in grain and leaves, respectively. The right part illustrate the architecture and distribution of HSEs in the promoter regions of each HSP90. One rectangle represents one HSE. For clarity, the length of the rectangle is tenfold the length of HSEs. “HMN” represents the varied HSEs that have mismatched nucleotide at specific position and have lager response magnitude than that of canonical HSEs.

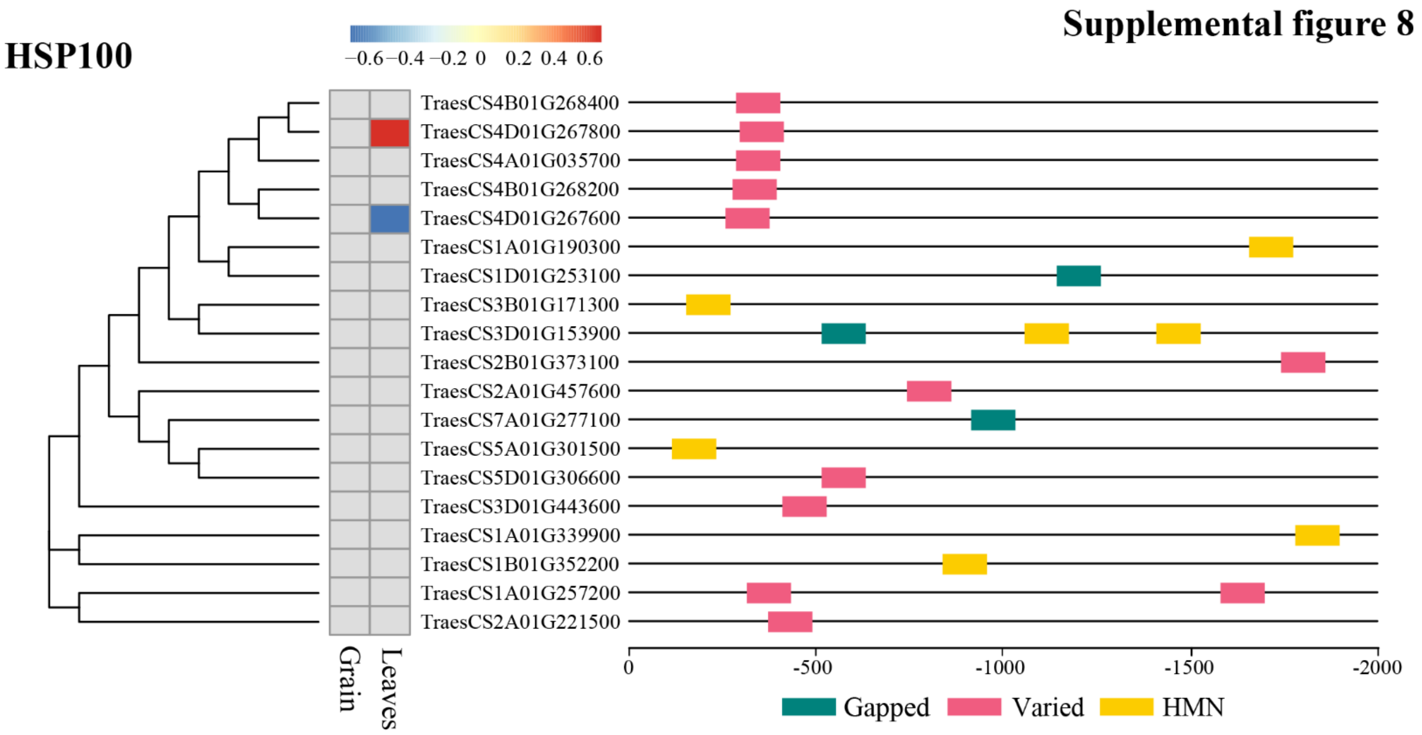

Supplementary Figure 8. The response magnitude and distribution of HSEs of HSP100 in grain and leaves.

The phylogenetic tree was generated based on the full length amino acid sequences of HSP100 with the neighbor-joining (NJ) method. The heatmaps on the left represents response magnitude of HSP100s in grain and leaves, respectively. The right part illustrate the architecture and distribution of HSEs in the promoter regions of each HSP100. One rectangle represents one HSE. For clarity, the length of the rectangle is tenfold the length of HSEs. “HMN” represents the varied HSEs that have mismatched nucleotide at specific position and have lager response magnitude than that of canonical HSEs.

Supplementary Figure 9

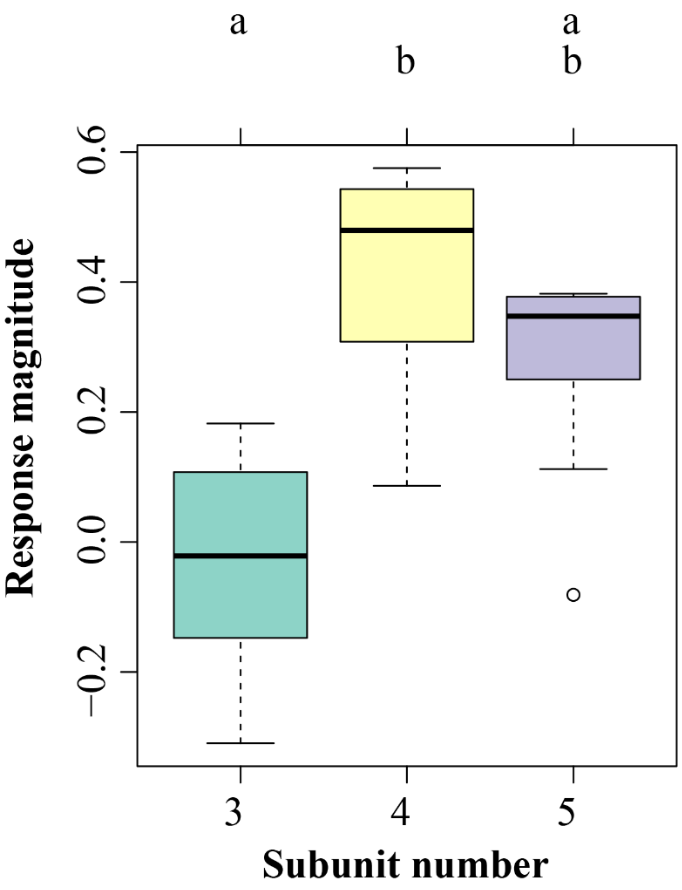

Supplementary Figure 9. The response magnitude of TVGs in leaves.

The x-axis represents the subunit number of HSEs and the y-axis represents the response magnitude of TVGs in leaves. The letters above the boxplot represents the level of significance and the data sets with different letters were significantly different at the level of *p-value* < 0.05.
